# Determinant factors for chronic kidney disease after partial nephrectomy

**DOI:** 10.18632/oncoscience.393

**Published:** 2018-02-23

**Authors:** Oscar D. Martín, Heilen Bravo, Marcos Arias, Diego Dallos, Yesica Quiroz, Luis G. Medina, Giovanni E. Cacciamani, Raul G. Carlini

**Affiliations:** ^1^ Clínica Cooperativa de Colombia, Universidad Cooperativa de Colombia - Facultad de Medicina, Villavicencio, Colombia; ^2^ Servicio de Nefrología y Trasplante Renal, Hospital Universitario de Caracas, Caracas, Venezuela; ^3^ Hospital Metropolitano de Santiago (HOMS), Santiago, República Dominicana; ^4^ Department of Urology, University of Verona, Verona, Italy; ^5^ Fundacion Universitaria Ciencias de la Salud Hospital de San Jose, Bogotá, Colombia

**Keywords:** partial nephrectomy, chronic kidney disease, kidney-sparing surgery, prognostic factor, predicting factor

## Abstract

The objective of this review is to evaluate the factors that determine the development or deterioration of Chronic Kidney Disease (CKD) after partial nephrectomy (PN).

When current literature is reviewed, it is found that factors that influence renal function after partial nephrectomy, are multifactorial. Those are divided into pre-surgical factors, such as hypertension, diabetes mellitus, urolithiasis, obesity, metabolic syndrome among others; intra-surgical factors, like the surgical technique used, the remaining healthy tissue, the experience of the surgeon, the time and type of ischemia among others. Lastly, post-surgical factors, also impose some influence on the post-surgical renal performance.

It was also found that minimally invasive surgery, in addition to its known advantages, seems to offer a greater field of action in the future that will allow more nephrons preservation in any future surgical scenario.

Finally, the current trend is to perform PN on all patients, in whom surgery is technically feasible regardless of the approach used, without risking oncological outcomes, patient safety, and without being exposed to any additional complications.

## INTRODUCTION

Sparing most of the nephrons without compromising the oncological outcome has played an important role in the kidney malignancies management. Partial nephrectomy (PN) has the advantage of maintaining or preserving as much kidney function as possible, ensuring greater survival and reduced morbidity. On this matter, two interesting concepts were proposed recently: trifecta (negative surgical margins, no postoperative complications and warm ischemia time of ≤ 25 minutes) and pentafecta (trifecta components plus: preservation of more than 90% of renal function and no presence of chronic kidney disease).

The current trend to manage most renal masses using the PN technique or nephron-sparing surgery has provided a better understanding of chronic kidney disease (CKD) physiopathology in patients that underwent these surgical interventions. Consequently, PN has also allowed to expand the indications to more complex surgeries and, at the same time, ensuring to preserve as much healthy functional tissue as possible (1, 2).

In this article, we will review the pre, intra, and post-surgical factors that could have an impact on the kidney function.

## EVIDENCE ACQUISITION

**Figure 1:**
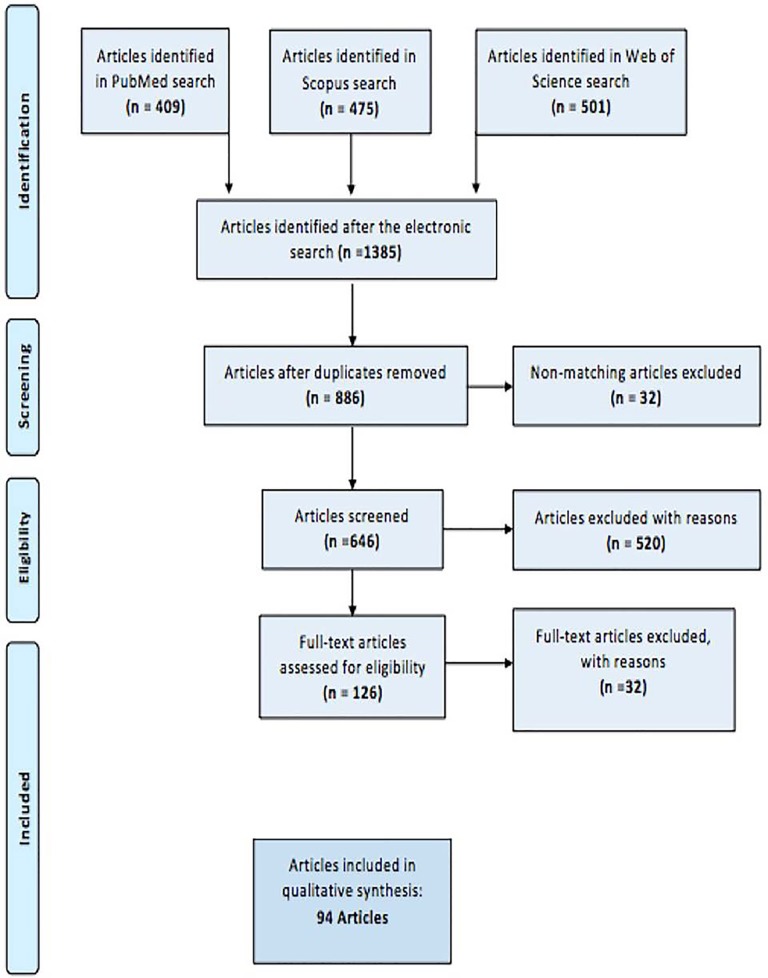
Flow chart of the identified study Research strategy.

We performed a systematic review limited to article in English language published from. A specific search on Pubmed, Web of Science and Scopus a databases included “partial nephrectomy” OR “kidney-sparing surgery” AND “chronic kidney disease” OR “chronic renal disease”. Editorial, commentary, abstract, reviews, book chapters, experimental studies on animal or cadaver were not included in the review. Three of us (OM, LM and GC) independently reviewed the literature using inclusion and exclusion criteria. Al disagreement about eligibility were resolved by a discussion until a consensus was reached. A total of 94 articles were identified for potential inclusion based on the review and were eligible for the qualitative analysis. This study was performed using guidelines set out by PRISMA (Preferred Reporting Items for Systematic Reviews and meta-analysis statement (3) (Figure 1.)

## EVIDENCE SYNTHESIS

### 1 Pre-surgical factors

Multiple nephrological and urological diseases can affect pre-surgical renal function. Nephrological diseases are typically the most common causes, such as diabetes mellitus, high blood pressure, obesity, and metabolic syndrome. On the other hand, urological diseases, which can also affect patients' baseline renal function, are represented by kidney stones, urinary tract infection (UTI), vesicoureteral reflux, and neurogenic bladder.

All of these conditions contribute to the development of CKD. Which is defined as kidney damage for >3 months (confirmed by pathological changes in biopsy samples or kidney damage markers, and proteinuria), with or without changes in glomerular filtration rate (GFR), or a GFR <60 ml/min/1.73 m2, during 3 or more months, with or without kidney damage (4). Nephrologic and urologic diseases are not the only risk factors for CKD; hypertension, obesity, and smoking pose an increased risk for CKD (RCC) (5). It is for this reason that CKD can be found in up to 26% of RCC patients with normal pre-surgical serum creatinine levels (6).For instance, A histologic evaluation of 110 patients, in whom radical nephrectomy was carried out, different histological changes were found on the tissues surrounding the tumor (such as vascular sclerosis, atheroembolic disease, and diabetic nephropathy (glomerular hypertrophy, mesangial expansion, and diffuse glomerulosclerosis. 6 months of follow-up after surgery, these patients experienced a significant decrease in the levels of renal function, when compared to those with normal tissue around tumor (7).

Another important aspect is that, in the past, urologists recommended radical nephrectomy to their patients, as the impact on renal function was minimal based on the data from several large cohort studies of living kidney donors. After long-term follow-up periods, these studies showed that after unilateral nephrectomy for transplant donation, the normal renal function could be maintained by a single kidney, and for this reason, radical nephrectomy was the gold standard for management of renal masses. However, the kidney transplant donor population differs significantly from RCC patients, because usually kidney donors are healthy and RCC patients often more comorbidities. .

The relevance of patients' baseline renal function lies in the fact that CKD stage G3a, G3b, G4 or G5 act as an independent predictor factor for postoperative renal function deterioration during the first 30 days (8). Also, It is clear that the previous renal function status plays a critical role in the risk for worsening of a preexisting CKD. For example, it has been shown that in patients with solitary kidney has also been seen that the pre-existing renal function is an important independent risk factor for stage G5 CKD and hemodialysis in future (9).

Although partial nephrectomy (PN) in patients with CKD stages G1, G2 and G3 is associated with a minimum decrease in renal function compared with patients without CKD; they are at increased risk for surgical complications and longer hospitalization time (10). Some studies suggest that despite the high complications risk, robotic partial nephrectomy technique affects kidney function in patients with pre-existing CKD marginally (11).

### 2 Surgical factors

The surgical factors that tend to affect outcomes the most are time, type of ischemia, the surgical technique used, the complexity of the case, and the amount of parenchyma preserved

#### 2.1 Time and type of ischemia

**Figure 2:**
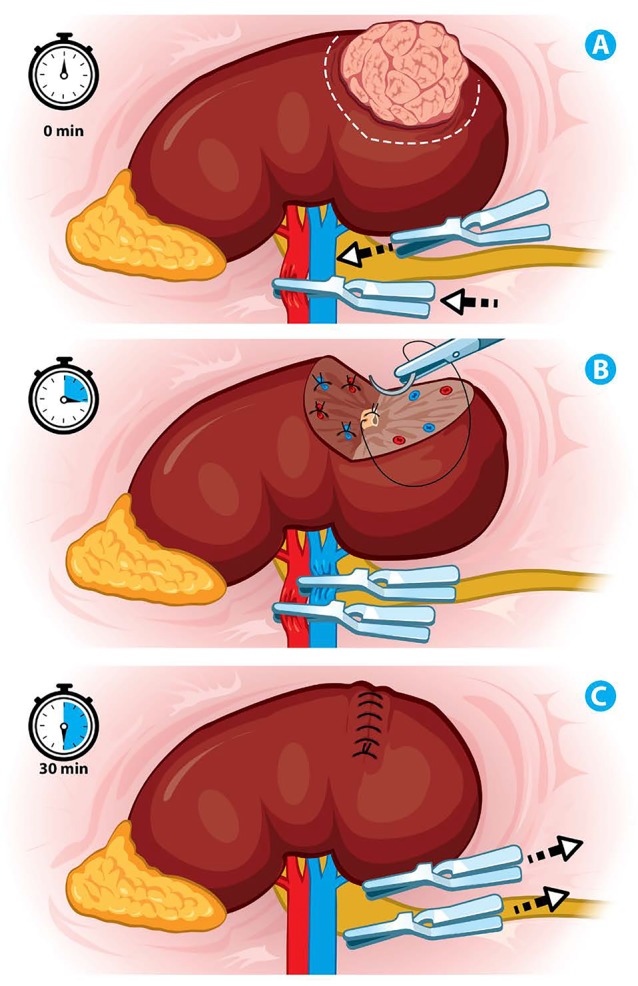
Ischemia techniques (A) Renal artery and vein clamping. (B) Selective closure of the collecting system and renal vessels. (C) Renorrhaphy, withdrawal of laparoscopic bulldogs with total time of warm ischemia of 30 minutes.

Time and type of ischemia are crucial factors associated to nephron-sparing surgery outcome. The ideal type and duration of ischemia that would ensure a better long-term renal function has not been established. However, period of 20-30 minutes has been proposed as the safest ischemia time to avoid irreversible renal parenchymal damage.. (Figure 2)

The first studies reported that complete renal function recovery was linked to ischemia time. Recovery of renal function is complete within minutes after 10 minutes of ischemia, hours after 20 minutes, 3 to 9 days after 30 minutes, several weeks after 60 minutes, and incomplete or absent after 120 minutes of ischemia. (12)

Notwithstanding, there is controversy on this topic because the current knowledge of renal ischemia is derived mainly from animal studies, renal transplant and retrospective human studies, that report conflicting data regarding the response and tolerance of the human kidney to ischemia (12, 13). To this date, there is only one prospective study with 40 patients who underwent renal biopsies after 30 minutes of ischemia and analyzed results showed that an ischemia time of 30-60 minutes could be safe with subsequent minor structural changes without a severe functional loss (13).

In an analysis of 362 patients with solitary kidney, the proposed ischemia time was a little less than 20 minutes and the maximum 25 minutes. After this period, for each extra clamping ischemia minute, between 5% and 6% of risk of renal damage is added (odds ratio: 1.05 for each minute with a p <0.001. In single kidneys with pre-existing CKD, the risk of acute renal failure is increased by 7% for each additional minute. (14)

The type of ischemia is related to the use of warm or cold ischemia. Cold ischemia allows the operator between 35 to 58 minutes of renal artery clamping without functional compromise. Presumably, this increasing in the ischemia time is because of a diminished kidney's metabolism, stabilization of the pH in tissues, among others, which leads to a hypothetical decreased damage to the renal parenchyma on ischemic conditions (15). (Figure 2)

However, it has been suggested that no matter what type of ischemia is being used, as much as the total time of ischemia along with the quantity and quality of preserved renal parenchyma. These results were demonstrated by the analysis of 660 and 1396 patients. This multivariate analysis showed that the remaining kidney tissue was an independent factor for subsequent renal function (16, 17).

#### 2.2 Surgical technique

Nowadays, the options available for the management of renal masses are open, the minimally invasive approaches (laparoscopic and robotic), percutaneous ablative therapies and active surveillance.

Initially, when partial nephrectomy technique was introduced, radical nephrectomy was considered the gold standard, but subsequent studies demonstrated the superiority of PN taking into account quality of life and renal function, with the same oncological long-term results, while avoiding the cardiovascular risk posed by radical nephrectomy. (18)

Thereafter, in an effort to decrease associated morbidity to surgical procedures laparoscopic, robotic, ablative techniques have been proposed (i.e. cryoablation, and high intensity focused ultrasound) along with active surveillance as management alternatives. (19)

**Figure 3:**
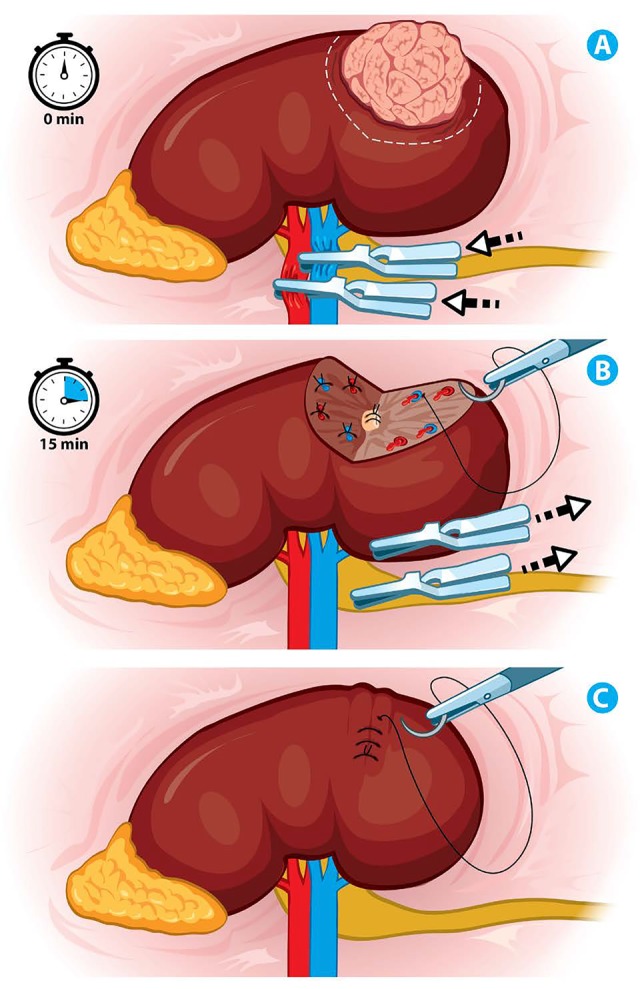
Ischemia techniques (A) Renal artery and vein clamping. (B) Selective collecting system and renal vessels closure, withdrawal of laparoscopic bulldogs in 15 minutes. (C) Renorrhaphy without clamping.

Renal ischemia is still the most controversial discussion. The “Early Unclamping technique” appeared in an attempt of decreasing the ischemia time to the minimum. A 100 patients series showed better results in reducing the recommended average ischemia time by 50% from 30 minutes to 14 minutes (p <0.0001) (Figure 3), This is achieved performing an initial parenchymal suture under total ischemia, with the remaining renorraphy with a revascularized kidney resulting in subsequent renal function improvement (p <0.0003). However, there is an increased risk of major bleeding, with increased number of complications and the possibility of re-clamping the hilum, which would add greater parenchymal injury due to reperfusion syndrome (20). (Figure 3)

**Figure 4:**
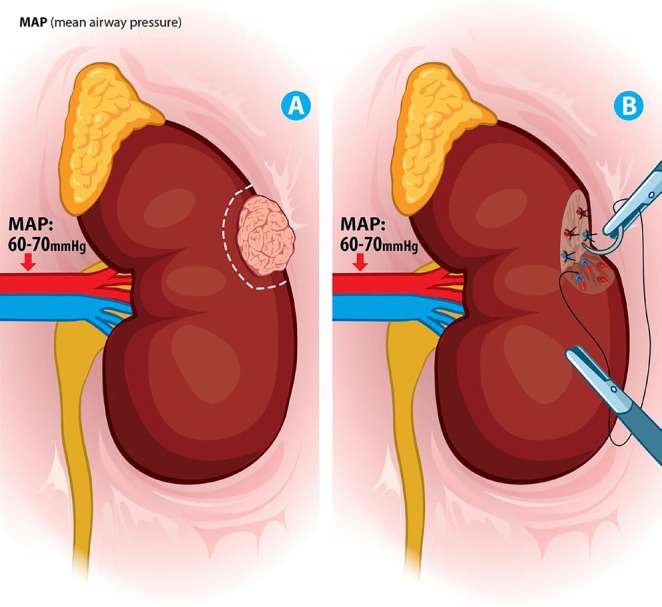
(A) Tumor lesion in the middle renal segment with controlled hypotension MAP of 60-70 mmHg, (B) complete resection of tumor lesion with minimal renal parenchymal bleeding Direct hemostatic control with absorbable points.

In the intent to looking for even better results, the “zero ischemia” technique was developed, which refers to not clamping the renal hilum; the initial experience was of 15 patients only. The procedure was used in cases of exophytic masses of size not exceeding 7 cm, being more viable in those of size <4 cm demonstrating its feasibility and safety along with promising results in renal function (21). Later, controlled hypotension techniques were developed to reduce bleeding during removal of the mass and closure of renal parenchyma, along with trans-arterial selective embolization as an alternative to improve the bleeding and technique itself (22). (Figure 4)

**Figure 5:**
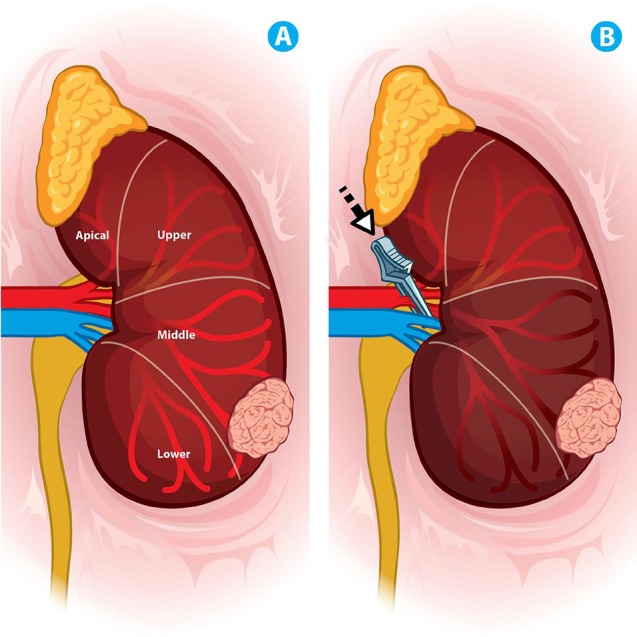
Zero ischemia technique (A) Anterior left renal artery distribution, renal mass on the inferior pole with middle artery feeding. (B) Middle arterial branch selective clamping.

Despite this novel concept, it could not be applied in all cases due to the risk of peri-operative bleeding and complications (23). In order to conserve the benefits of off-clamp procedures without the bleeding risk, the “selective and supraselective clamping technique” was described (24). (Figure 5). These methods require dissection of the branches of the renal artery until fourth generation branches with the purpose of reaching the feeding artery of the tumor and preventing damage to the rest of the renal parenchyma. These techniques are implemented along with the use of indocyanine green dye for the proper identification of tumor's feeding artery as well as the healthy tissue, achieving better short-term renal functional outcomes than with traditional renal artery clamping (25). It is important to point out that the application of these techniques on masses located in the Brodel line constitutes the major drawback because in these cases there are many branches of the feeding arteries.

Recent discussions have taken place regarding the best option in PN; which have been focused on the comparison between the two types of zero ischemia techniques (off-clamp and selective clamping), against clamping of the renal artery in 162 patients. In the previously mentioned study, the renal function in the short term was superior in the two zero ischemia techniques compared to the approach of clamping of the renal artery (p = 0.04) after three months. However, six months after surgery, no statistically significant difference was seen in the outcome of renal function between zero techniques ischemia and renal artery clamping as long as the ranges of warm ischemia time were 20-30 minutes. (26)

Nowadays, PN is the gold standard for T1a (<4 cm) masses, (16) and it is widely used for T1b (4 cm to 7 cm). This is because of the fact oncologic results are equivalent to radical nephrectomy with the addition of the improved kidney function and cardiovascular risk reduction. Also, PN has been succeeded in T2 (> 7 cm) masses with satisfactory technical, oncological and functional results (27).

Robotics has shown to be superior to the laparoscopic approach in terms of less bleeding and shorter warm ischemia time (28) while expanding the indication for endophytic tumors, for example (1). In addition to this, PN technique is limited by the surgeon's skills. In a multivariate analysis of 660 patients, experience was found it to act as an independent factor. A 116 procedures study series comparing the learning curve between robotic and laparoscopic approaches found that the decreasing in the percentage of glomerular filtration rate in the postoperative period was lower in the robotics group (29). Also, complexity, and size of the tumor appears to be important, assessed by the classic nephrometry scores and the recently proposed ones, as main predictors of the postoperative renal function. (30)

Another important factor for the surgical management of renal malignancies is the method used for bleeding control and closure of the renal parenchyma. In a study of 15 patients, a comparison was made between PN without cortical reparation (only a running, base layer closure of collecting system and vessels) versus conventional closure (base layer closure plus a running sliding clip cortical renorrhaphy); this resulted in less ischemia time on the non-renorrhaphy group (12 minutes versus 20 minutes). A surprising finding was observed in the follow-up period (more than four months) after comparing pre and post-surgery percentage of kidney volume loss with tomography, resulting in 9cc without renorrhaphy and 17cc with renorrhaphy. This finding was suggested to occur as a consequence of the hypoperfusion/constriction of the parenchyma margins when the cortical renorrhaphy is performed (31).

#### 2.3 Residual renal parenchyma

The amount of healthy remaining kidney tissue post-PN is now considered to be the most important factor for future renal function (13). Although there are no recommendations for the amount of the healthy kidney tissue that must be conserved, before making the decision of undertaking a radical nephrectomy, we can say that the higher the remaining healthy tissue, the better the outcome for further renal function, and longer intervals of time before chronic kidney disease ensues; that will require renal replacement therapy in the future (10). However, it has been recommended to preserve at least more than 50% of the healthy tissue. Otherwise, is very likely to develop glomerular hyperfiltration syndrome causing focal segmental glomerulosclerosis, which leads to ERC in the future. (32).

In single kidney patients (with or without CKD) who develop renal cell carcinoma, treatment requires a careful balance between considerations for the malignant disease and the maintenance of renal function. However, the main stem should always be to preserve as much parenchyma as possible without jeopardizing oncologic outcomes.

### 3 Post-surgical factors

The factors that determine CKD after the procedure are pre-surgical and surgical factors. After the PN, patients must control their underlying pathologies, besides modifying their lifestyle, improving their nutritional habits, along with appropriate monitoring in the future.

## CONCLUSION

Renal function after PN is multifactorial and depends on pre-surgical factors (comorbidities and previous renal function state). Moreover, intra-surgical aspects such as the technique used, ischemia technique or renal mass preservation along with proper follow-up.

Minimally invasive surgery appears to offer broader therapeutic scope for the renal masses without compromising oncological outcomes un proper hands.

Finally, it seems that robotic PN is technically feasible, without risking oncological outcomes, renal function, and patient safety and without exposing to additional complications.
